# Quantum Key Distribution with Displaced Thermal States

**DOI:** 10.3390/e26060488

**Published:** 2024-05-31

**Authors:** Adam Walton, Anne Ghesquière, Benjamin T. H. Varcoe

**Affiliations:** School of Physics and Astronomy, University of Leeds, Leeds LS2 9JT, UK; dr_aghes@yahoo.fr (A.G.); b.varcoe@leeds.ac.uk (B.T.H.V.)

**Keywords:** thermal states, quantum key distribution (QKD), experimental, continuous variables, correlation, computing

## Abstract

Secret key exchange relies on the creation of correlated signals, serving as the raw resource for secure communication. Thermal states exhibit Hanbury Brown and Twiss correlations, which offer a promising avenue for generating such signals. In this paper, we present an experimental implementation of a central broadcast thermal-state quantum key distribution (QKD) protocol in the microwave region. Our objective is to showcase a straightforward method of QKD utilizing readily available broadcasting equipment. Unlike conventional approaches to thermal-state QKD, we leverage displaced thermal states. These states enable us to share the output of a thermal source among Alice, Bob, and Eve via both waveguide channels and free space. Through measurement and conversion into bit strings, our protocol produces key-ready bit strings without the need for specialized equipment. By harnessing the inherent noise in thermal broadcasts, our setup facilitates the recovery of distinct bit strings by all parties involved.

## 1. Introduction

Traditionally, optical communication has been the cornerstone of quantum key distribution (QKD), relying on proven technologies like lasers and optical fibers that are capable of transmitting information over long distances and achieving reasonable bit rates. However, optical frequencies can often be unwieldy and impractical for short-range applications, such as communication between mobile devices, medical implants, or electronic car keys and locks. This challenge primarily arises from the ‘last mile problem’, where difficulties in the alignment (the pointing accuracy) of a narrow laser source hinders efficient communication. An additional issue in the optical region involves the regular need for line-of-sight or a fiber connection between the relevant parties, which may be impractical or expensive to implement. Recognizing the significant gap between optical QKD and the practical demands of short-distance communication, a recent shift in focus has been directed towards exploring microwave QKD [1,2]. These varying use cases are already seen in communication systems in which microwave and radio links are preferred methods for short-range communication, while high-speed optical fibers excel in linking hubs [1]. This is also seen at a smaller scale with Internet connectivity, where optical fibers are used to connect to housing, with WiFi providing connections from a router to mobile devices.

Quantum communication has witnessed remarkable strides [3,4] in the last few years, starting with the launch of a quantum satellite in 2016 that has recently been used to transmit quantum keys over 1200 km [5]. This marked a significant step towards realizing global-scale quantum key distribution (QKD). However, the last couple of years have also witnessed a number of other efforts to explore QKD in a range of frequency regimes [1,2,6,7,8,9,10,11,12,13,14].

Recently, it has been recognized that a thermal state is a useful resource for QKD [13,14,15,16,17,18,19,20,21,22], and this project aims to develop a practical system to enable thermal-state QKD. Thermal radiation is useful because it exhibits ‘bunching’, resulting in high levels of noise correlation. This correlation gives rise to quantum discord [23], which has been long recognized as a necessary condition for QKD [24]. However, one of the problems with a thermal state is the transmission of the state to the receiver. By their very nature, thermal states lack coherence, and even though the thermal photon numbers in the microwave are large, a microwave thermal state on its own is not capable of maintaining a significant signal strength for transmission.

The primary goal of this research is to experimentally demonstrate the use of thermal states as a resource for QKD. In this paper, we introduce a new approach where we use displaced thermal states. Unlike conventional thermal states, which are centered at zero amplitude and have uncertain phase, displaced thermal states acquire the phase of the displacing coherent state while retaining the noise characteristics of the thermal state. The security for continuous-variable QKD comes from vacuum noise fluctuations. The equivalent noise in a thermal state can be orders of magnitude larger than that of an equivalent coherent state, making displaced thermal states an attractive target for secure communication.

Over the last few years, we have been developing a theoretical background for thermal-state quantum key distribution [14,15,16,17]. In this sequence of papers, we have shown that Hanbury Brown and Twiss correlations form a resource for QKD [17], we have created a protocol for exchanging keys using thermal states [15], we have demonstrated the security of the protocol [16], and we have shown that thermal states can be exchanged with displaced thermal states [14]. At the same time, we have seen the development of passive-state QKD [18,19], which has been following a similar path.

The outcome of this research is to show that QKD is in the reach of current communications equipment. In this paper, our goal is to show that this novel thermal resource can be harnessed for secure communication, and by presenting this, we hope to contribute to making communication more accessible and reliable. The setup we employ has the advantage of allowing us to use off-the-shelf radio equipment, and in the case of long-range transmissions, free space broadcasts could also allow us to remove the need for a waveguide, which is costly to put in place.

### Protocol

The protocol for thermal-state QKD was presented in Ref. [15] together with a security proof. In this protocol, we require a source that emits thermal radiation that can be detected by both legal parties and the eavesdropper. This protocol has been demonstrated to be secure when the eavesdropper uses an entangling cloner [15]; however, as an entangling cloner has not yet been devised, we have performed the protocol using a classical eavesdropper (purely as a demonstration). Nevertheless, we will see that classical Eve has a similar performance to the optimal quantum eavesdropper. This is a natural consequence of the nature of a thermal source. Moreover, while the circumstance in which the source is not trusted [16] has been considered, for the purpose of this demonstration, the source will remain under Alice’s control.

The formal statement of the protocol is:

- Alice creates a beam from a trusted thermal source.
- Alice uses BS1 to divert part of the signal to her detector and sends the rest to Bob.
- During the transmission to Bob, Eve can intercept some or all of the signal (BS2).
- The bunched nature of the pairs coming out of BS1 means that fluctuations present in amplitude measurements at Alice’s detector are correlated with those at Bob’s detector.
- To derive their data, Alice and Bob slice these fluctuations as convenient; as an example, a fluctuation above the median could be a 1 and a fluctuation below the median could be a 0.
- Like any QKD scheme, our protocol requires quantum correlations. To confirm that the measurements by Alice and Bob are correlated, they verify the thermal nature of their signal. Thus, Alice sends Bob small chunks of data for them to perform a $g^{\left(2\right)}\left(0\right)$ calculation. $g^{\left(2\right)}\left(0\right)>1$ means that the signal is thermal.
- Alice and Bob now have a stream of independent and randomly correlated bits from which they can derive a key, the security of which they can improve with cascade and advantage distillation, as per any QKD scheme.

From Bob’s (and Eve’s) perspective, the outputs of a thermal source resemble those of sources used in a Gaussian-modulated coherent-state (GMCS) protocol [18,25]. This protocol involves coherent states drawn from a Gaussian distribution and broadcasted by Alice. The statistical similarity allows us to apply the same security proofs valid for GMCS QKD to the thermal state protocol, including allowance for finite key effects [17,26] and composable security [11,26,27].

For the purpose of the current paper, we use a pair of commercial off-the-shelf radio transceivers, which can be connected by waveguides or using free space connections. Waveguides have the benefit where we can eliminate or significantly reduce external factors such as atmospheric attenuation and signal dispersion, allowing us to isolate and study the inherent characteristics of the broadcast. This controlled environment allows us to gain insights into the behavior of the thermal-state QKD protocol without the confounding effects of external interference.

Free space transmission presents additional challenges such as losses and interference and additional noise sources; nevertheless, we have achieved short-range broadcasts. It is worth noting that short-range free space transmission is not a limitation; in fact, short-range QKD would be well suited for applications like payment systems, car remotes, Wi-Fi, and Bluetooth, where communication typically occurs within a limited distance. In these scenarios, the challenges of atmospheric attenuation and signal dispersion are less pronounced, making short-range free space transmission a practical and efficient choice.

## 2. Quadrature Phase-Shift Keying

**Figure 1:**
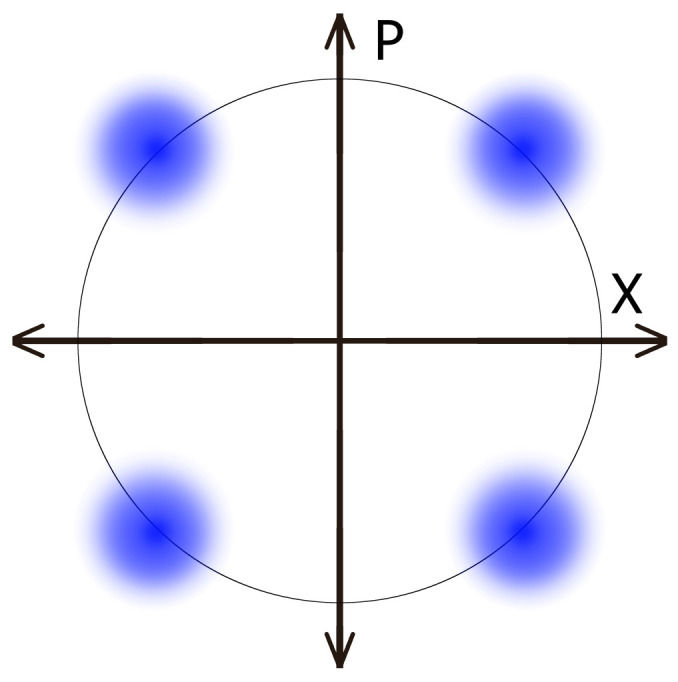
QPSK. A form of PSK, quadrature phase-shift keying is where two bits of information are sent per signal through the assignment of one of the four possible combinations of two bits to each of the four clusters. The axes refer to the amplitudes of a pair of sinusoidal waves, which differ in phase by $\frac{\pi }{2}$. These amplitudes are adjusted to produce different signals. While this will not be used for encoding our actual key, a broadcast of this form assists in correcting timing problems.

For ease of broadcasting, we use the classical communication system known as quadrature phase-shift keying (QPSK). This is a modulation scheme widely used for transmitting digital data over radio frequencies. While this will be a useful element of our protocol, the key itself will not be encoded in this manner. QPSK represents a set of techniques where data are encoded by varying the phase of a carrier wave among four possible values, typically 0°, 90°, 180°, and 270°. Each of these phase values corresponds to a different symbol (see Figure 1). There are four states; hence, this allows for the transmission of two bits per symbol.

Using the language of quantum optics, we can also describe QPSK in terms of displaced thermal states. A displaced thermal state arises when a thermal state undergoes displacement by a coherent state. A thermal state describes a system in thermal equilibrium with its surroundings, characterized by a distribution of energy levels following a thermal distribution. In the context of QPSK, we can draw an analogy to displaced thermal states by considering the phase diagram commonly used in quantum optics. In this diagram (Figure 1), the horizontal axis represents the real part of the amplitude of the quantum state, while the vertical axis represents the imaginary part. Each point in this phase space corresponds to a unique quantum state.

Now, let us consider the four phase shifts used in QPSK modulation: 0°, 90°, 180°, and 270°. Each of these phase shifts can be viewed as a displacement of a thermal state by a coherent state with a specific phase difference. For example, a phase shift of 0° corresponds to a normal displacement, and adding a π/2 phase shift to the carrier corresponds to displacement by a coherent state with a phase difference of 90°.

**Figure 2:**
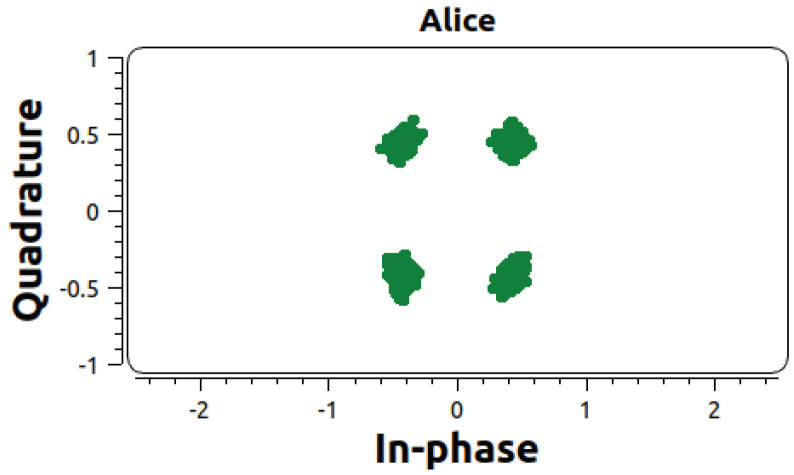
Thermal clusters. A snapshot of the output of the thermal source after modulation. Using GNURadio, a constellation modulator produces the four clusters expected in QPSK, as shown in Figure 1.

By encoding data using different phase shifts, QPSK effectively manipulates the quantum states in phase space, allowing for the transmission of digital information. This type of encoding is the current standard for digital communications systems. Therefore, it becomes relatively straightforward to produce and measure a displaced thermal state with high accuracy. Figure 2 shows a measurement of four displaced states using an off-the-shelf QPSK receiver.

**Figure 3:**
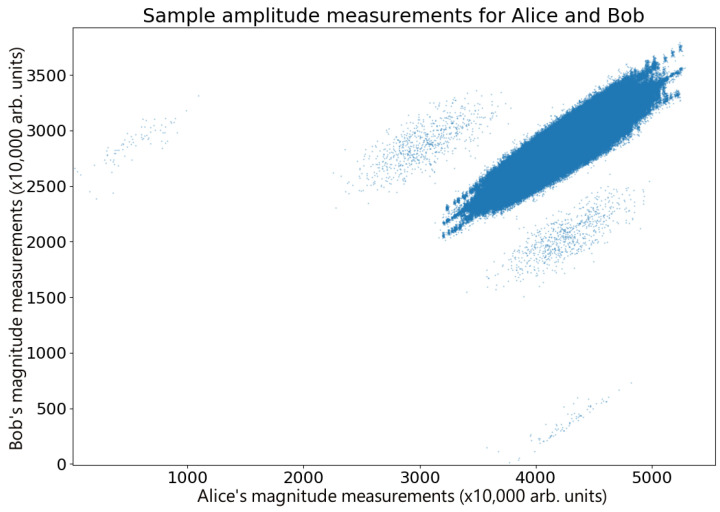
Correlations in thermal states. A comparison of a sample of amplitude measurements performed by Alice and Bob after adjusting for time errors, displaying Hanbury Brown and Twiss correlations. The data display a number of correlated overlapping features, each with a high level of correlation. Small phase hops occur between the source and the Bob–Eve beam splitter, probably due to thermal changes in the waveguide, giving rise to random phase drifts. For short times, the phase remains stable, and this can be seen as drifting correlation. In addition to this are stray reflections in Alice’s transmission line, which create faint ghost images that can be seen on either side of the main peak. These have very little effect on the overall correlation because the ghost reflections only represent a tiny fraction of the data.

The benefit of QPSK encoding is that binary data can be encoded as ‘quadratures’. Four quadratures provide two bits of binary information. This allows us to ‘error correct’ the transmission phase. The transmission of a random binary code leads to a unique unraveling of the phase sequence, which therefore allows Alice and Bob to align their data. This is a critical component of thermal-state QKD, because they must be able to locate the Δt=0 peak in the $g_{2}$ correlation spectrum [17]. The unique phase unraveling therefore allows them to uniquely locate the peak correlation (see, for example, Figure 3).

**Figure 4:**
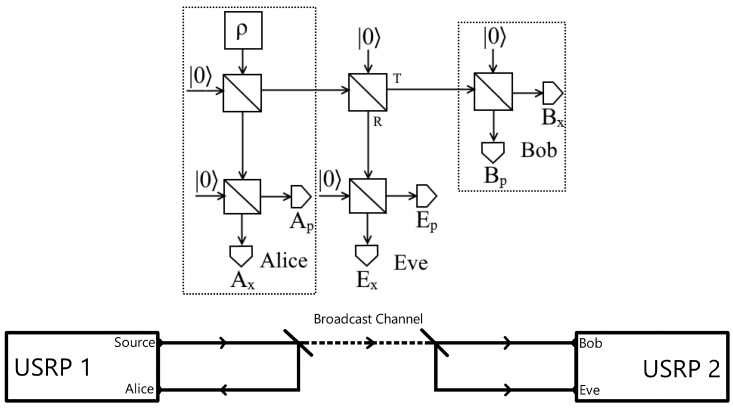
Method. A diagram of the central broadcast thermal protocol (top) accompanied by an experimental setup (bottom). A beam from a thermal source is divided between Alice and a broadcast channel. Eve intercepts Bob’s beam with a splitter of transmittance T, with the other splitters being 50:50. Each party performs heterodyne measurements to find quadrature values of their received broadcast. A photograph of Alice and Bob’s setup is shown in Figure A1. We perform this through using a pair of USRPs (Universal Software Radio Peripherals), one of which sends an initial broadcast towards a power splitter to be partially directed back into another channel representing Alice. The second output of this splitter is the broadcast channel, leading to a second power splitter representing Eve’s interception. Each of the outputs of this second power splitter leads to separate channels on a second USRP, representing Bob and Eve. The first diagram was originally used in ’Thermal state quantum key distribution’ [14] and is licensed under CC-BY 4.0.

Having established the nature of our broadcast signal, the method employed in this study follows a well-established framework for thermal-state quantum key distribution (QKD) and its variant using displaced thermal states [14,28]. A simplified flow chart illustrating the communication process is provided in Figure 4. A displaced thermal source is directed onto a beam splitter, with the resulting output channels connecting to Alice and Bob, who aim to establish secure communication. Before Bob can perform any measurements, Eve attempts a beam splitter attack. Each party subsequently uses heterodyne detection, repeatedly measuring pairs of quadrature values to obtain a series of correlated measurement pairs, denoted as $\left(x_{i},p_{i}\right)$. These measurements are then processed to derive correlated bit strings by computing $z_{i}=\sqrt{x_{i}^{2}+p_{i}^{2}}$ for each measurement pair. We use a coarse-grained slicing method where a binary value of 0 or 1 is assigned to each $z_{i}$ value based on whether it falls above or below the median value obtained at that detector. It should be reiterated that the specific QPSK cluster that each measurement was derived from is not involved in this calculation. When creating the bit strings to be refined into keys, we are only concerned with amplitude measurements, and this is not a discrete [29,30] QKD protocol.

There are two protocols that we could use to transmit continuous quantum information: a ‘prepare-and-measure protocol’, where Alice generates random Gaussian states and transmits them to Bob; and the thermal [15,17] or passive-state QKD protocol [18,19]. From Bob’s perspective, the two protocols are identical due to the statistical equivalence between the Gaussian states transmitted by Alice and the passive states used in the passive-state QKD protocol.

From Bob’s point of view, in both scenarios, he receives quantum states that exhibit Gaussian statistics. These states are characterized by their mean and variance, which encapsulate information about the transmitted quantum information. In both cases, Bob’s task is to perform measurements on the received quantum states to extract the relevant information for key generation. While in the prepare-and-measure protocol, Bob measures the received Gaussian states using compatible measurement bases, typically chosen randomly. He then records the measurement outcomes and communicates with Alice to establish a shared secret key through classical post-processing techniques.

Similarly, in the passive-state QKD protocol, Bob receives passive states generated by Alice, which also exhibit Gaussian statistics. Bob performs measurements on these received states and follows the same procedure as in the prepare-and-measure protocol to extract the shared secret key. Overall, despite differences in the physical implementation of the two protocols (active-state generation in prepare-and-measure versus passive-state generation in passive-state QKD), from Bob’s perspective, the statistical properties of the received quantum states are identical. This equivalence allows Bob to employ the same measurement and key generation procedures in both scenarios, resulting in similar operational outcomes for the two protocols. With this in mind, there are security proofs over a wide range of performance metrics that support the concept of a thermal state resource [13,16,18,19,20,22,31,32].

**Figure A2:**
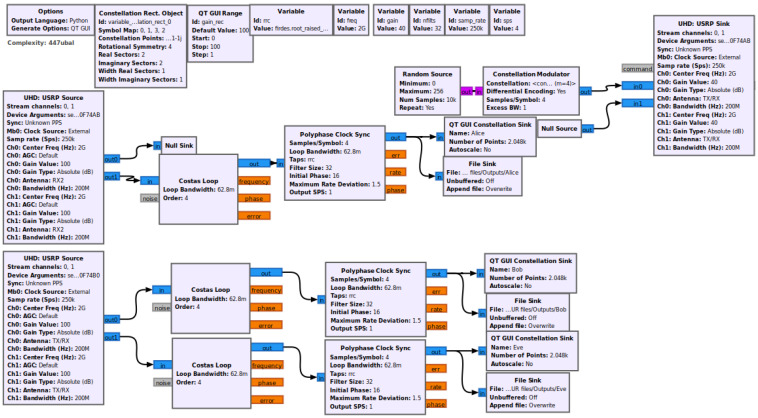
The GNU Radio flowchart. Signal processing performed by GNU Radio. Four clusters are sent from a source to Alice and Bob for amplitude measurement. These are randomly generated for each broadcast and fed to a constellation modulator, which produces the desired form of broadcast. One USRP controls the initial broadcast on a transmitting channel and Alice on a separate receiving channel. Meanwhile, the second USRP acts as Bob and Eve by utilizing two separate receiving channels, applying some time corrections before recording data.

To perform the experiment, we use USRP-2901 radio transceivers broadcasting at a frequency of 2 GHz using a PRS10 Rubidium oscillator as a time reference, and a GNU Radio is used for signal processing, with the signal processing flowchart being shown in Figure A2. We use Costas loops and polyphase clock sync blocks to stabilize the phase and assist in synchronizing the measurements.

The first experimental results were obtained using waveguides between Alice, Bob, and Eve. This has two primary effects. The first is that we can be sure that all of the signal that does not go to Bob goes to Eve (this is not possible in a free space apparatus), and it therefore conforms with the standard requirements for testing the security of CVQKD [25,33,34]. Secondly, it provides a very low phase noise environment to establish the operational parameters.

The two main limitations with the waveguide channel are as follows: firstly, the broadcast signal is attenuated to avoid sending large signals into the receivers (maximum input signal is −30 dBm, or 1μW), and secondly, thermal variations during the measurement lead to phase and amplitude fluctuations as a result of small changes in the length of the waveguides. The effect of this can be seen as a phase ‘hopping’ in Figure 3.

The free space channel uses an omni-directional antenna at both the transmitters and the receivers side; this means that there is no specific directionality to the signal, and this allows Bob to be located anywhere around Alice. This means that neither Eve nor Bob has a specific advantage. However this comes at the expense of substantial signal losses limiting the range of the transmission.

## 3. Experimental Results and Security Analysis

The constellation modulator produces four displaced states that are equally spaced around a circle centered on the origin in phase space. A snapshot of the received QPSK broadcast is shown in Figure 2. We processed quadrature measurement results by rotating the angles of the four QPSK elements so that they overlap, effectively creating a single displaced state.

**Figure A3:**
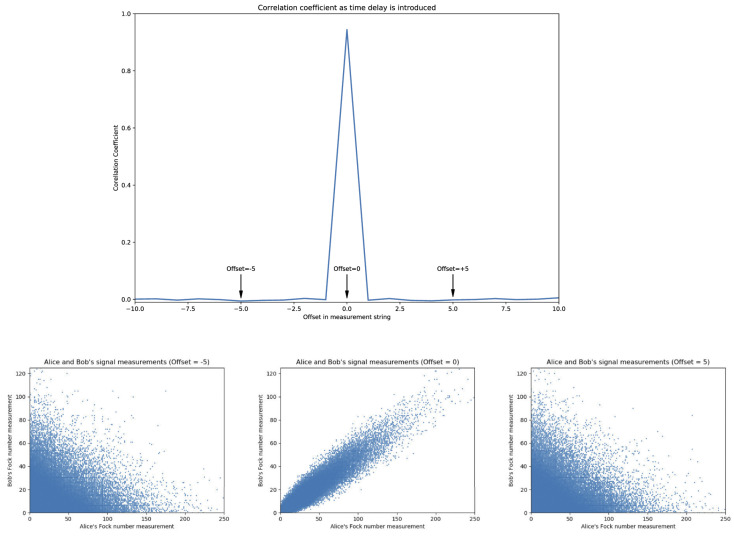
Thermal State Correlations. A demonstration of the correlations inherent in the outputs of a splitter with thermal input. Using QPSK to synchronize Alice and Bob’s amplitude measurements means that those amplitude measurements will be correlated. This diagram was originally used in ’Thermal state quantum key distribution’ [14] and is licensed under CC-BY 4.0.

However before we could accomplish this, we needed to establish a time synchronization between the sender and receiver. A 2 GHz signal has a wavelength of 15 cm; hence, there can be several oscillations of 2π between the transmission and reception, essentially randomizing the phase relationship between Alice and Bob. Therefore, in order to synchronize the measurement times, Alice and Bob compare the digital signals obtained by observing the quadrant phase. The string of measurement results that were obtained presented a unique signature of the timing, and this allowed Alice and Bob to correct for time delays. Revealing the alignment of the phase quadratures to the eavesdropper does not affect security as the secret key will be derived using correlated signal noise. This problem of correctly synchronizing the measurement times is the reasoning behind arranging the thermal states in the format of a QPSK broadcast rather than a single displaced thermal state. From previous work (reproduced in Figure A3) [14], we see the utility of including the QPSK aspect into this protocol in that the timing corrections provided by it reveal the highly correlated intensity measurements between the involved parties, which we require to produce appropriate bit strings.

**Figure A4:**
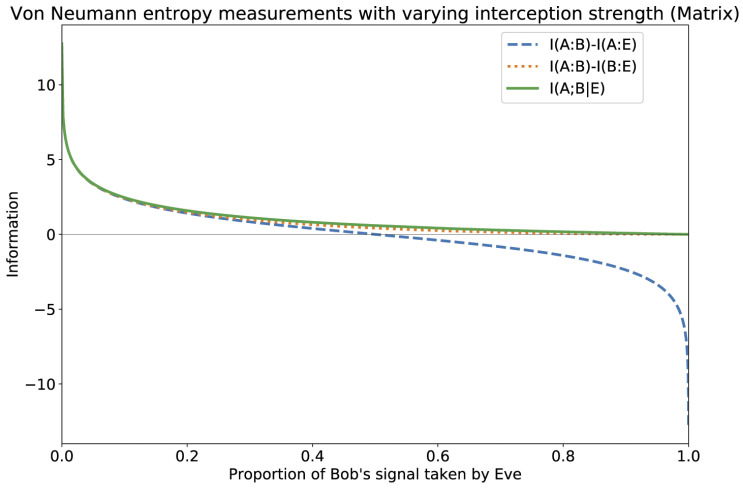
Von Neumann Analysis. An earlier analysis of the effect of loss on the von Neumann mutual informations between each party. This diagram was originally used in ’Thermal state quantum key distribution’ [14] and is licensed under CC-BY 4.0.

Once the time delay is accounted for, bit strings are derived from the correlated amplitude measurements. From these bit strings, Shannon mutual information I(X;Y) are calculated, which are used to test if the protocol is successful. For a key to be distilled from the bit strings after employing advantage distillation, we require that the conditional mutual information $I\left(A;B|E\right)$ is greater than zero [35]. We have previously [14] seen that, from a von Neumann analysis of the system, this requirement was maintained regardless of loss in the system on the channel leading to Bob and Eve. This has also been reproduced in Figure A4.

Also of note concerning secrecy requirements is work performed on a similar protocol involving thermal broadcasts by Qi, Evan, and Grice [18]. An observation here was that key rates increased with average photon number count with simulations performed up to 500. Given radio equipment broadcasts with power far in excess of this, amplitude measurements will be provided in arbitrary units.

**Figure 5:**
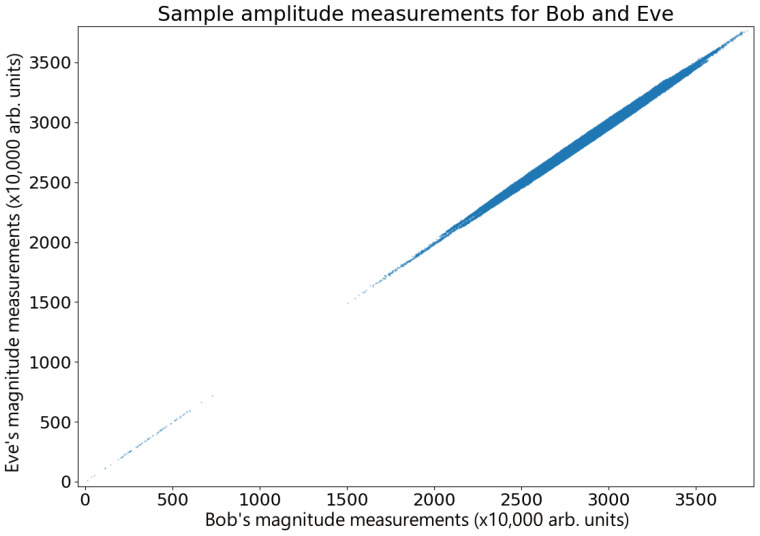
Waveguide measurements for Bob and Eve. A comparison of a sample of amplitude measurements performed by Bob and Eve after adjusting for time errors. As there is no practical difference between Eve and Bob, there are therefore fewer amplitude errors, and the long duration signal is highly correlated due to the higher degree of symmetry between their respective detectors.

For continuous data, it is possible to calculate the $g_{2}$ correlation [17]; however these data are discrete, making the $g_{2}$ inaccessible. Figure 3 shows a plot comparing Alice and Bob’s amplitude measurements in a sample set of data ($n=3\times 10^{6}$ points) after compensating for time delay errors. These measurements are highly correlated (r = 0.9264), clearly displaying the correlations expected for the Hanbury Brown and Twiss effect, with Bob and Eve’s measurements showing the same behavior (r = 0.99362), as shown in Figure 5. For the data presented here, $I\left(A;B|E\right)=0.04688$ and $\Delta I=I\left(A;B\right)-I\left(B;E\right)=0.18154$. While this specific example meets the standard QKD requirements for key exchange [25,33,34], shot-to-shot variability in the measurements resulting from thermal drift in the waveguide (see Figure 3) means that this is not always the case as ΔI occasionally fluctuates into negative numbers. However, the combination of advantage distillation and privacy amplification ensures that Alice and Bob are able to retrieve a key in general. Therefore, given that the waveguide tests showed some inconsistencies, we transitioned to a free space broadcast channel for more reliable results, which in any case is the more realistic scenario for secure key exchange.

**Figure 6:**
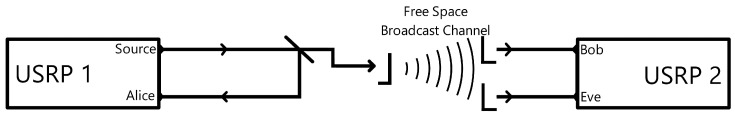
The free space apparatus. The adjusted version of the experimental setup to allow for wireless broadcasting. As in the wired setup shown in Figure 4, the first USRP remains connected to a power splitter, which sends part of the broadcast back into a separate input for Alice. A whip antenna is connected to the second output of the power splitter, replacing the broadcast channel that was previously connected to Bob and Eve with free space. A pair of receiving antenna are connected to separate channels in the second USRP, giving Bob and Eve wireless methods of receiving the broadcast.

The experimental setup was adjusted so that the output from Alice’s thermal source is now connected to a whip antenna, which broadcasts an omni-directional signal that can be detected by Bob and Eve. Alice still uses a waveguide to her local receiver in this model, in agreement with the assumption that Alice is in control of the source. This arrangement is shown in Figure 6.

A whip antenna typically exhibits an omni-directional emission pattern in the horizontal plane, meaning that it radiates electromagnetic waves uniformly in all directions around the antenna axis; hence, there is no longer a preferred direction, and Bob and Eve are free to move around the source. The downside of this is that the cross-section of Bob’s antenna is relatively small; therefore, he experiences much higher loss. However, as we are comparing physical measurements performed by the eavesdropper to those performed by Bob, the free space setup makes security less challenging owing to the reduction in the correlations between Bob and Eve’s measurements. To some extent, this situation lies outside of Eve’s control. Here, we are following a ’realistic’ eavesdropper scenario [36], noting that it is unreasonably obvious for the eavesdropper to try to collect more of the signal than Bob over a small distance.

We performed a free space broadcast over a distance of 1 m at a sample rate of 250 k samples per second. The limited range was a result of the amount of reflective surfaces in the lab, the source power, and antenna configurations. As noted above, this is still a practical range for several applications. Moreover, the number of reflective surfaces reduce substantially when the device is used ‘outdoors’, allowing for a greater range in practice. As with the previous setup, no error correction is employed beyond compensating for time delay and the phase shifts added during the protocol.

**Figure 7:**
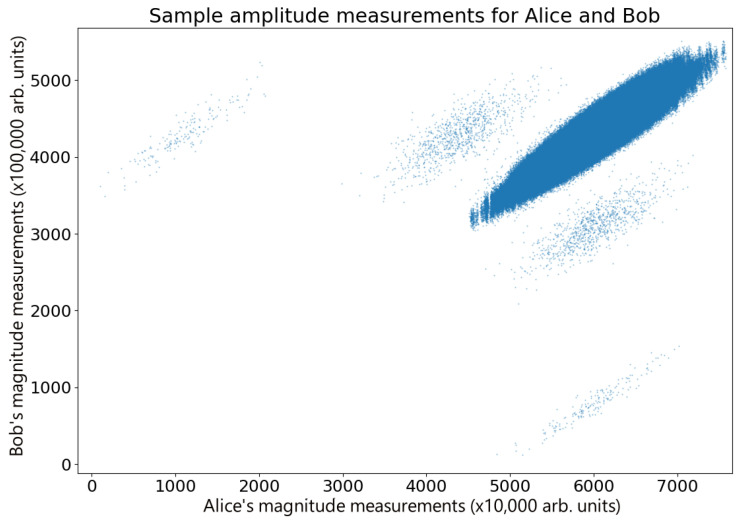
Correlations in thermal states. A sample (*n* = 3,000,000) of amplitude measurements performed by Alice and Bob. The measurement results were less correlated than the waveguide version displayed in Figure 3; however, they were still suitable for key distribution. The amplitude measurement results are shown in Figure A5. Again, ghost reflections can be seen in the data similar to the waveguide model and are therefore most likely a result of an impedance mismatch in Alice’s apparatus.

**Figure 8:**
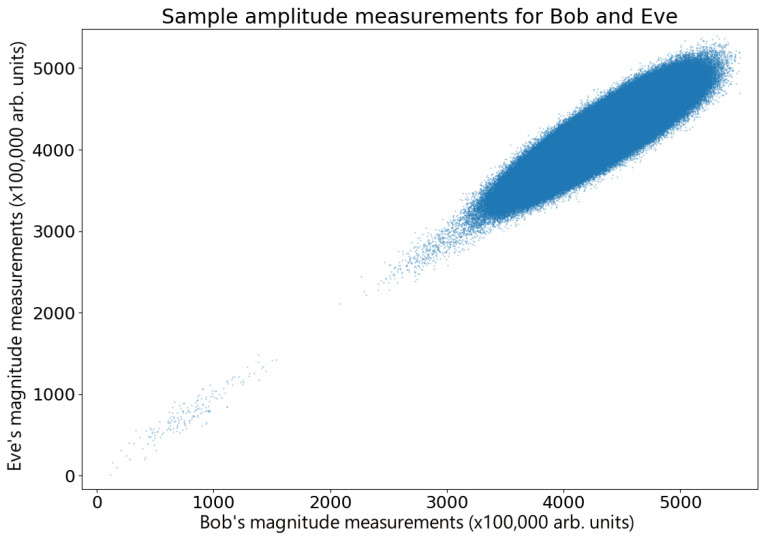
Free space measurements for Bob and Eve. A sample (*n* = 3,000,000) of amplitude measurements performed by Bob and Eve. The additional loss results in less correlated measurements.

Free space broadcasts in general displayed higher variance than in the waveguide version; however, bit strings produced through this method were still suitable for conversion into keys with reverse reconciliation. Comparing Alice and Bob’s measurements shows very similar behavior in both the waveguide and free space setups, as seen in Figure 7. However, a major impact of the change in free space was the decrease in correlation between Bob and Eve, dropping from near-identical bit strings down to a mean correlation coefficient of r=0.89. A comparison of a sample of such measurements are shown in Figure 8.

Additionally, we see faint copies of the measurement cluster repeated at several other points around the plot. This is likely due to multipath propagation, in which the receiver detects copies of the broadcast signal that have been reflected by other objects and is a source of error, which is difficult to completely remove. This is a well-known problem in free space communication, and its detection here is not surprising. There are multiple ways to reduce the impact of this, such as through repeated hopping of broadcast frequencies [37]; alternatively, in more controlled circumstances, RF-absorbing material can be used to reduce reflections. While this effect does negatively impact the performance of a free space broadcast, it should be noted that Eve would be similarly affected due to also having no control over such reflections.

The free space variant was repeated over a distance of 1 m, producing 20 sets of bit strings with approximate lengths of $3\times 10^{6}$ bits each (12 s of data). From this, we calculated mutual information values and correlation coefficients, giving a mean conditional mutual information $I\left(A;B|E\right)$ of 0.126±0.046. As this is a positive value, we have achieved a sufficient condition for secure communication [38].

For direct reconciliation, we found $I\left(A;B\right)-I\left(A;E\right)\approx 0$. This is expected due to the symmetry in the system. For reverse reconciliation, we found $I\left(A;B\right)-I\left(B;E\right)=0.082\pm 0.06$. This meets the conditions for secret key exchange. We found a bit error rate of approximately 11.3%±2.9% between Alice and Bob.

We have therefore demonstrated the use of displaced thermal states as a resource for quantum key distribution in a microwave field using a ‘Passive State QKD’ configuration with QPSK assisting in timing corrections.

We have presented a simplified and feasible approach for secure communication, particularly in scenarios requiring short-range applications, which demonstrated the practical implementation of thermal QKD.

An interesting outcome is that this potentially enables the direct integration of QKD into existing communication systems, rendering it accessible and viable for real-world deployment. The QKD component and digital communications components are (almost) independent of one another with digital error correction, increasing the alignment of the states and therefore improving the secret key rate.

Looking ahead, avenues for exploration include extending the range of free space QKD and leveraging error-correcting codes to enhance performance. Our implementation of QKD within an off-the-shelf digital communication system exploits the analogy between QPSK modulation and displaced thermal states, creating a relatively seamless integration between quantum and classical communication protocols. This interplay provides valuable insights for future developments in the field and paves the way for further innovation.

## Figures and Tables

**Figure A1:**
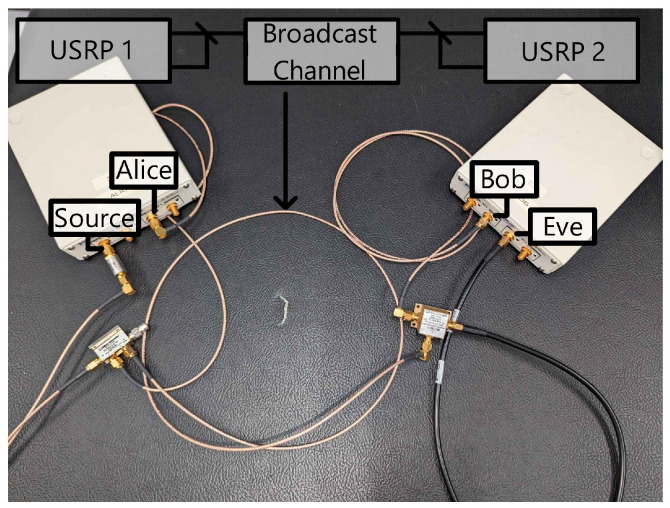
Experimental Setup. The experimental setup for a wired broadcast between Alice, Bob, and Eve. The first USRP sends the broadcast to a power splitter, which splits the signal between a separate input for Alice and a broadcast channel leading to the second USRP. An eavesdropper is added through splitting the broadcast channel, allowing the second USRP to act as both Bob and Eve through the use of two separate input channels. For wireless implementations of the protocol, the wired broadcast channel is replaced with a whip antenna and free space, with a pair of receiving antennae connected directly into the second USRP for Bob and Eve. Alice’s portion of the setup is unchanged.

**Figure A5:**
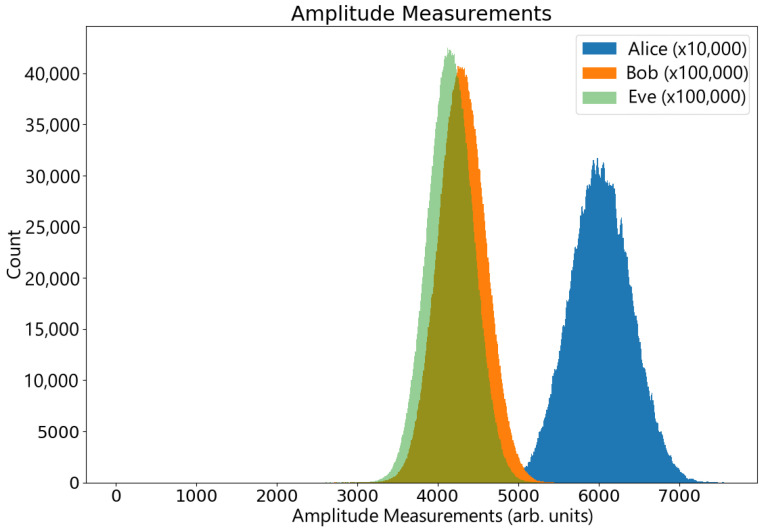
Free space thermal states broadcasting. A sample (*n* = 3,000,000) of recorded amplitude measurements used in Figure 7 and Figure 8. This more clearly displays the Gaussian form of the broadcast.

## Data Availability

The data used to plot the graphs in Figure 3, Figure 5, Figure 7 and Figure 8 are available at the DOI https://doi.org/10.5518/1521.
